# Optical Properties of Submillimeter Silver Nanowires Synthesized Using the Hydrothermal Method

**DOI:** 10.3390/ma12050721

**Published:** 2019-03-01

**Authors:** Michał Ćwik, Dorota Buczyńska, Karolina Sulowska, Ewa Roźniecka, Sebastian Mackowski, Joanna Niedziółka-Jönsson

**Affiliations:** 1 Institute of Physical Chemistry, Polish Academy of Sciences, 01-224 Warsaw, Poland; mcwik@ichf.edu.pl (M.Ć.); dbuczynska@ichf.edu.pl (D.B.); erozniecka@ichf.edu.pl (E.R.); 2 Institute of Physics, Faculty of Physics, Astronomy and Informatics, Nicolaus Copernicus University, 87-100 Torun, Poland; sulowska@fizyka.umk.pl; 3 Baltic Institute of Technology, 81-451 Gdynia, Poland

**Keywords:** silver nanowire, hydrothermal synthesis, peridinin-chlorophyll-protein, fluorescence microscopy, plasmonic enhancement

## Abstract

We report on the synthesis of long silver nanowires using the hydrothermal method, with H_2_O_2_ as the reducing agent. Our approach yields nanowires with an average diameter and length of about 100 nm and 160 µm, respectively, reaching the maximum length of 800 µm. Scanning electron microscopy (SEM) measurements revealed the presence of a thick, inhomogeneous poly(vinylpyrrolidone) (PVP) layer covering the nanowires, which with time becomes much more uniform, leading to well-defined extinction peaks in the ultraviolet-visible (UV-Vis) spectra. This change in morphology is evidenced also by the fluorescence enhancement behavior probed using protein complexes. Wide-field and confocal fluorescence microscopy measurements demonstrate strong, 10-fold enhancement of the protein emission intensity, accompanied by a reduction of the fluorescence decay time. In addition, for the aged, one-month-old nanowires, the uniformity of the intensity profile along them was substantially improved as compared with the as-synthesized ones. The results point towards the importance of the morphology of plasmonically active silver nanowires when considering their application in enhancing optical properties or achieving energy propagation over submillimeter distances.

## 1. Introduction

Metallic nanowires, characterized with diameters of a few hundreds of nanometers and lengths of tens of microns, can interact with light in a unique way. On the one hand, they exhibit the ability to be a plasmonic antenna and focus an electric field down tens of nanometers [1,2,3,4]. In addition, they can be viewed as plasmonic waveguides [5,6,7,8,9] which sustain propagation of surface plasmon polaritons (SPPs) over their lengths. The antenna concept has been utilized in metal-enhanced fluorescence [1,3,4,10], with implementations involving pigment-protein complexes [3,4], and for SERS substrates [11], while waveguiding in metallic nanowires has been used in signal processing or remote sensing [9,12,13]. In particular, the applications based on the energy waveguiding of metallic nanowires would require larger lengths of the nanowires accompanied with the ability to propagate SPPs over such distances. Distant SPP propagation can be achieved with nanowires that are defect-free, as structural defects, together with polycrystalline structure, are sites of energy dissipation. 

Various methods have been applied to synthesize silver nanowires (AgNWs) [6,14]. Lithographic methods can be used to produce structures of well-defined dimensions, however they frequently exhibit poly-crystalline structure and rather rough surfaces for SPP propagation that would result in substantial scattering, thus reducing propagation lengths. Hard template methods, where nanowires are synthesized in cylindrical membrane pores, like porous anodic aluminum oxide [15] or carbon nanotubes [16], yield structures with specified dimensions, but—similarly to the lithographic methods—are characterized by rough surfaces and a substantial degree of poly-crystallinity. Furthermore, since fabrication and the removal of templates is rather inefficient, the AgNWs yield is low, and the quality of the structures is not adequate for many applications, soft template methods have gained much interest. In the soft template methods shape-directing capping agents, such as cetyltrimethylammonium bromide (CTAB) [17] and poly(vinylpyrrolidone) (PVP) [18], are used to direct the crystal growth. Nanowires produced with these methods are single-crystalline with smooth surfaces and uniform widths, however control over dimensions of the AgNWs is limited, in particular in regard to their lengths. The polyol method, in which ethylene glycol is used as both the solvent and reducing agent, has recently become one of the most commonly used approaches to obtain AgNWs, mainly due to the simplicity and reasonable control of the morphology [19,20,21,22]. The diameters of synthesized nanowires are typically in the range of 50–200 nm, while their lengths are of 30–80 µm. In this respect, other approaches have been developed aimed at obtaining even longer nanowires. In comparison with the polyol method, the hydrothermal synthesis, where instead of ethylene glycol water is used as solvent, [23,24,25] allows for fabrication of AgNWs with average lengths exceeding 100 µm. Among the reducing agents that have been tested in the hydrothermal synthesis are glucose [24], ethylene glycol [25], and sodium citrate [26], while microwaves can be applied instead of heating to induce the AgNWs growth process [27].

The effect of metal enhanced fluorescence (MEF) [28] is associated with the ability of metallic nanostructures to focus the electric field of an incident light in their close vicinity (optical antennas), thus increasing spontaneous emission rates (Purcell effect) and/or excitation rates of fluorophores placed nearby. MEF yields higher emission intensities [29] and may result in improved photostability, and reduction of fluorescence decay time. The enhancement competes frequently with fluorescence quenching associated with energy absorption of the metal [1,28]. There are multiple factors, which determine the outcome of the interaction between metallic nanoparticles and fluorophores, including material, geometry, relative orientation and distance of the emitter with respect to the plasmonic structure, excitation wavelength, etc. Although ensemble experiments provide less detail of the nature of the MEF as compared to those carried out on single emitters [3] they are still useful in imaging of the local electric field [30], as well as SPP propagation lengths [31] using far-field microscopy. In contrast, near-field methods provide better spatial resolution and can be used to image in detail the mode propagation in plasmonic waveguides [32].

The aim of this work is to study plasmonic interactions between long AgNWs and protein complexes. This can be viewed as the first step in obtaining plasmonically active AgNWs with lengths that would allow remote control of the optical properties of any emitters at distances reaching millimeters. The nanowires used in this work were synthesized using a hydrothermal method, where H_2_O_2_ and PVP were used as a reducing agent and growth stabilizer, respectively. The lengths and diameters of the nanowires, assessed with bright-field optical microscopy and scanning electron microscopy (SEM), respectively, are in the range of 10–800 µm and 40–180 nm. SEM images indicate that the AgNWs, as synthesized, are covered with a thick but inhomogeneous PVP capping layer. This inhomogeneity results in variations of plasmonic interactions with emitters surrounding the nanowire, and thus in their optical properties. We find that over time the thickness of the PVP layer becomes more uniform, with significant improvement of the distribution of emission intensity along AgNWs. Importantly, the AgNWs facilitate plasmonic enhancement of peridinin-chlorophyll-protein (PCP) complex fluorescence intensity for a wide range of excitation energies, as shown using wide-field fluorescence microscopy, as well as the results of fluorescence dynamics. Our findings point out the importance of considering and understanding the morphology of AgNWs that can be applied for plasmonically-enhanced remote activation of optically sensitive nanomaterials. 

## 2. Materials and Methods

Synthesis of AgNWs was based on the approach presented in [24]. Silver nitrate (0.02M, 7,5 mL) (Sigma-Aldrich, St. Louis, MI, USA, 99.9999%), hydrogen peroxide (0.13 M, 2.5 mL) (Stanlab, Lublin, Poland, 30%), poly(vinylpyrrolidone) (PVP, Mw ≈ 40 000, K = 30) (0.5 g, 2.5 mL) (Sigma-Aldrich) and sodium chloride (0.03 M, 7.5 mL) (Fluka, Buchs, Switzerland, 99.99%) were prepared in deionized (DI) water (Elix, Millipore, Burlington, MA, USA, >15 MΩ/cm) as separate solutions. PVP was prepared at 65 °C, while other solutions were prepared at room temperature. During the synthesis the hydrogen peroxide was added to silver nitrate solution, and afterwards PVP and sodium chloride solutions were added. The grey-coloured suspension obtained was shaken and transferred to a 50 mL teflon-lined stainless steel autoclave reactor. The reactor was heated to 100 °C (4 °C/min) in oven and kept in this temperature for 1 h. Afterwards, the reactor was heated up to 165 °C (4 °C/min) for 24 h. After that the autoclave was slowly cooled to room temperature and the final product in the form of grey white precipitate was collected by centrifugation (300 rpm, 20 min) and washed three to five times with DI. The final product was dispersed in DI water to a total volume up to 3 mL and stored in a refrigerator (4 °C) for further use.

AgNWs lengths were evaluated using a bright-field (reflection image) mode of LV150 optical microscope (Nikon, Tokyo, Japan, 10× objective), using a Fi-color CCD camera (Nikon) for nanowires dispersed on a glass coverslip. Sample was prepared by depositing 4 µL of 500-fold diluted AgNWs in DI water. Scanning electron microscope (SEM) images were acquired for AgNWs on ITO substrate—a droplet (2 µL) of 500-fold or 50-fold diluted water dispersion of AgNWs was deposited on ITO and left to dry. Measurements were performed using a Nova NanoSEM 450 (FEI, Hillsboro, OR, USA) SEM microscope at 10 kV voltage in immersion mode. 

Ultraviolet-visible (UV-Vis) spectra of AgNWs and PCP solutions were taken at room temperature using Evolution 300 dual-beam spectrophotometer (Thermo Electron Corporation, Waltham, MA, USA) with a 10 mm optical path PMMA cuvette. Extinction spectra of aged AgNWs were collected using a Varian-Cary 50 spectrophotometer (Agilent Technologies, Santa Clara, CA, USA). An emission spectrum of PCP was collected using Fluorolog 3 (Jobin Yvon, Longjumeau, France) spectrofluorometer. Solutions were diluted 50-fold with DI water before taking spectra.

Fluorescence microscopy and spectroscopy were performed on samples composed of PCP complexes mixed with AgNWs in a poly(vinyl alcohol) (PVA) polymer layer. The sample was prepared by spin-coating an aqueous mixture of 4 μg/mL PCP (PerCP Streptavidin, BD Biosciences, San Diego, CA, USA), AgNWs diluted 50-fold, and 0.05% (5 μg/mL) PVA polymer (Moviol 20-98, Sigma Aldrich) on a glass coverslip. Distilled water was used to dilute all of the solutions. The resultant sample is a thin layer of PVA polymer, in which PCP complexes and AgNWs are suspended.

As a fluorescent reporter of plasmonic interactions, PCP photosynthetic complex was used [3,4]. The PCP complex features wide excitation range, from 400 to 650 nm, due to very efficient energy transfer between pigments within the complex [33]. Emission of the complex at 673 nm originates from the chlorophyll molecules [34], while the broad PCP absorption allows for substantial coupling between electronic states of the pigments within the PCP and plasmonic excitations in AgNWs over a wide wavelength range. 

The optical properties of PCP and AgNWs mixtures were studied using confocal and wide-field fluorescence microscopy. The confocal microscope allows for measuring emission spectra, as well as collecting fluorescence decay curves. It is based on the Nikon Ti-S inverted microscope body. Samples are illuminated by a BDL-SMN488 laser (Becker and Hickl, Berlin, Germany) operating in a pulsed mode, with 20 MHz repetition rate and 12 μW excitation power. Fluorescence emission spectra were collected using back-illuminated CCD detector (Andor iDus DV 420A-BV, Andor Technology, Belfast, Ireland) with an Amici prism used for light dispersion. Fluorescence decay curves were collected using id100-50 (ID Quantique, Geneva, Switzerland) avalanche photodiode coupled with a Time Correlated Single Photon Counting module (SPC-150, Becker and Hickl). Combination of HQ655LP (Chroma, Bellows Falls, VT, USA) optical filter and HQ675-20 (Chroma) bandpass filter was used for extracting fluorescence of PCP complexes. 

Wide-field microscopy experiments were carried out using a setup based on Nikon Eclipse Ti-U inverted microscope. Excitation at central wavelengths of 405, 480, 532, 630 nm was provided by a set of LED illuminators (Prizmatix, Holon, Israel). Excitation power was set to 100 μW for all wavelengths utilized in the experiments. Epi fluorescence setup employs Plan Apo, 100×/1.4 NA oil immersion objective (Nikon). Excitation and emission radiation is separated with T650LPXR (Chroma) dichroic mirror. A set of optical filters: FEL0650, FELH0650 and FB670-10 (Thorlabs, Newton, NJ, USA), was used at the input of iXon Du-888 EMCCD (Andor Technology, Belfast, Ireland) camera. Observation area is limited to around 55 µm × 55 µm, while amplification was set to 5-fold and collection time was set to 1 s in all experiments. 

## 3. Results and Discussion

**Figure 1:**
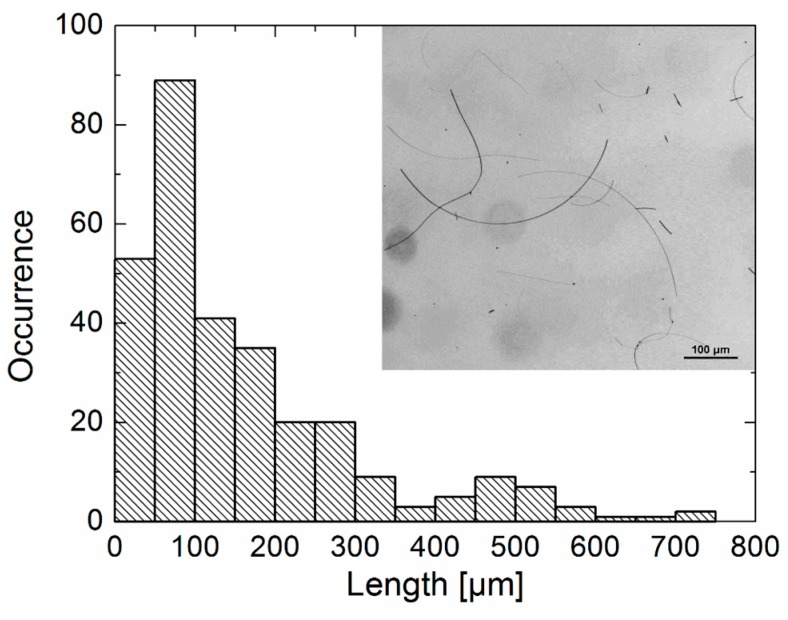
Distribution of the lengths of AgNWs after the synthesis, in the inset an example of the reflection bright-field image is shown.

The AgNWs were synthesized using a hydrothermal method with hydrogen peroxide. Based on previous work by Zhang et al. [35] the hydroxyl peroxide was applied to eliminate non-twinned particles and favor planar twinned seeds formation. In this way it is possible to obtain nanowires with almost no contamination with silver spherical nanoparticles and, in addition, with a large fraction of submillimeter-long AgNWs. Bright-field reflection images, as shown in the inset to Figure 1, were collected for AgNWs dispersed on a glass coverslip. We typically find nanowires of various lengths, with some spare silver nanoparticles. In Figure 1 we plot a distribution of lengths extracted for over 300 clearly resolved AgNWs. It reveals a broad range of the AgNW lengths with the average value of (161 ± 144) µm. Importantly, a significant percentage (10%) of AgNWs exhibits lengths over 400 µm, and since the nanowires are clearly visible in optical images, the longest ones can be isolated from all the others in a rather straightforward way. This can be done either by filtering out the dispersion after synthesis, or subsequently after depositing a suspension on a glass substrate.

**Figure 2:**
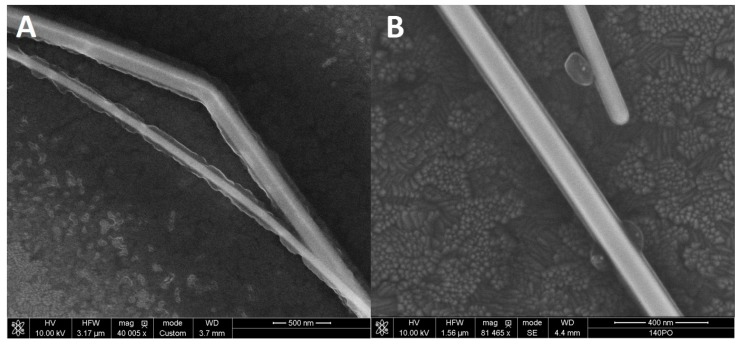
Scanning electron microscope (SEM) images of freshly synthesized (**A**) and aged (**B**) AgNWs.

To assess the morphology of AgNWs we collected a series of SEM images of nanowires dispersed on ITO substrate. Measurements were performed for as-synthesized nanowires, as well as 11 days after the synthesis. The results are displayed in Figure 2, where typical images of as-synthesized (Figure 2A) and aged (Figure 2B) AgNWs are compared. All of the nanowires are composed of a metallic core and a PVP polymer coating layer. For almost all of the studied nanowires, regardless of whether right after synthesis or those that were aged, we find no variation of the metallic core diameter over the length of a nanowire. The diameters of the nanowires, as extracted for over 60 individuals, were in the range from 40 to 180 nm, with the average value of 78 nm.

Furthermore, SEM measurements reveal a clear difference between the thickness of the PVP layer observed for as-synthesized and aged AgNWs. In the first case, the layer of PVP is very inhomogeneous along the nanowire. Indeed, its thickness varies from essentially zero, in the holes, up to 60 nm. This is probably due to a complex formation between PVP and traces of hydrogen peroxide [36]. As can be seen in the SEM image of aged AgNWs, these inhomogeneities disappear with time and, as the result, the PVP capping layer is generally very uniform along the nanowire. The thickness of the PVP layer for the aged AgNWs is typically around 10 nm.

Such a dramatic change in the morphology of AgNWs, in particular including the uniformity of the PVP capping layer, should have a strong impact on the plasmonic interactions with these structures; namely, the 10 nm-thick PVP layer measured for aged AgNWs should provide excellent conditions for obtaining fluorescence enhancement, as it can be considered a spacer inhibiting fluorescence quenching [1]. On the other hand, for AgNWs covered with such very inhomogeneous PVP layers as shown in Figure 2A, one should observe rather large variations of fluorescence signal, from low intensities, corresponding to the emitters that are very close to metallic surfaces and thus their emission is quenched, over some enhanced emissions in locations, where the PVP thickness is around 10–30 nm. 

**Figure 3:**
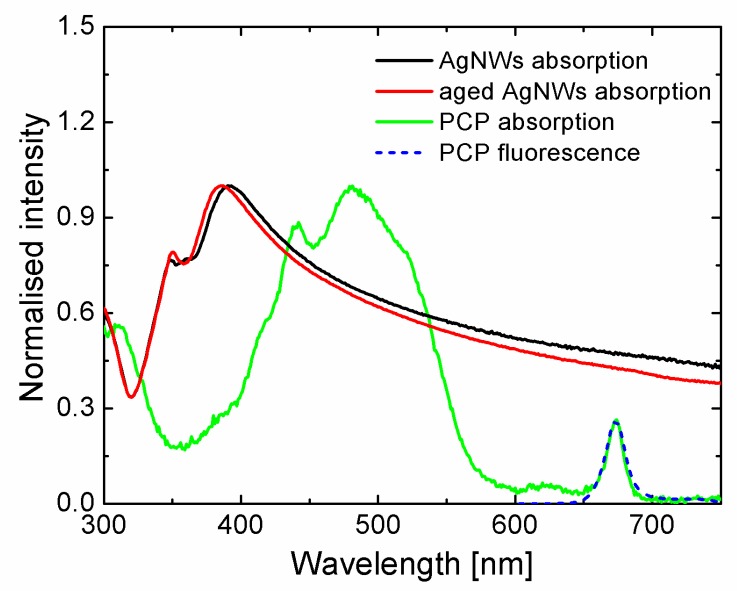
Extinction spectra of the same batch of AgNWs measured just after synthesis (black) and one month later (red), absorption (green) and fluorescence spectrum (blue) of aqueous solution of peridinin-chlorophyll-protein (PCP) complexes.

Absorption spectra of AgNWs and PCP solutions are shown in Figure 3. The PCP absorption spectrum is in agreement with the spectrum provided by the producer and previous results [33], with a maximum at 481 nm. The emission spectrum of the PCP solution features the maximum at roughly 673 nm, also in agreement with previous results. In the case of AgNWs, we collected extinction spectra both, directly after the synthesis and one month later. The spectra were measured for the same solution. The extinction spectrum of freshly synthesized AgNWs has a sharp peak at 393 nm, which can be attributed to transversal plasmon mode of the nanowires and some indistinguishable features around 360 nm, where absorption originating from quadrupolar modes should be located. The spectrum extends from maximum value at 393 nm towards lower energies and spans the entire range of visible radiation. In contrast, the spectrum measured after one month yields two narrower and more distinct peaks at 385 nm and 350 nm. Moreover, although the spectrum extends towards lower energies, the intensities are lower compared to the data obtained for the as-synthesized AgNWs. Taking into account the results of the SEM studies, this effect can be attributed to dissolution of the PVP cap and its more uniform distribution along the nanowires. In this way, the refractive index along AgNWs is also more uniform. Therefore, since the plasmon resonance frequency is sensitive to the refractive index of media, the smoothing of the PVP layer on AgNWs should result in refined extinction spectra of the aged AgNWs.

**Figure 4:**
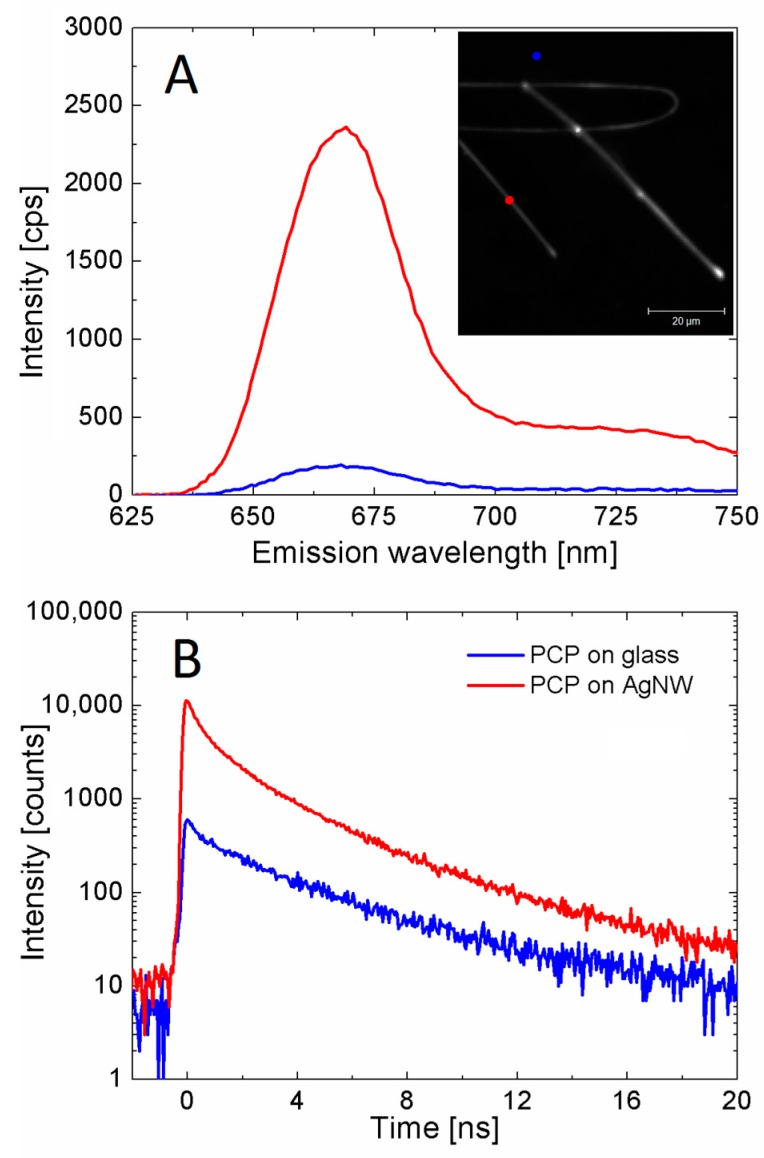
Emission spectra (**A**) and fluorescence decay curves (**B**) of PCP complexes, collected when the excitation laser spot is placed on a single AgNW position (red) and off any nanowire (blue). Neither spectra nor decay curves are normalized.

We apply confocal fluorescence microscopy to study the interactions in a sample composed of PCP complexes mixed with AgNWs. In the first step we collect a fluorescence intensity map, and next a series of spectra and fluorescence decay acquisition is carried out. An example of a fluorescence intensity map is shown in the inset of Figure 4, and it reveals the presence of three bright elongated shapes with increased intensity compared to the background, where the PCP complexes were also present. Importantly, the positions of these elongated shapes correlates with locations of nanowires observed in transmission mode for the same sample area. Therefore, the brighter emission observed in Figure 4 can be attributed to the MEF effect for PCP complexes placed in the vicinity of the AgNWs. The spectra and decay curves were measured for several locations across the map, and in Figure 4 we compare the results obtained when focusing the laser on the nanowire (red) and off the nanowire (blue). 

Both fluorescence spectra and corresponding decay curves indicate the increase of fluorescence intensity, which is accompanied with shortening of the decay time. The emission intensity reaches its maximum at around 673 nm, which is similar to native PCP. In the case of the emission spectra, the enhancement is of the order of tenfold, which is quite remarkable taking into account that in addition to the PCP complexes coupled with plasmon excitations in the AgNWs, many uncoupled PCP complexes are probed when the excitation spot is focused on a nanowire. There are some shifts in fluorescence spectra positions likely due to differences in the local environment, unlike in previous experiments [3,4]. This can be due to thinner PVA layer used in this work, which leads to higher proportion of PCP complexes being affected either by plasmon excitations in AgNWs, or effects associated with glass-polymer or polymer-air interface. 

Ten more sets of emission spectra and fluorescence decay curves were collected for PCPs in close proximity of AgNWs and for PCPs away from the AgNWs. The results follow the trend seen in Figure 4, indicating much higher emission intensity for PCP complexes placed in the vicinity of AgNWs as compared with those placed on a glass coverslip. In order to evaluate the interaction between PCP complexes and AgNWs, we fitted fluorescence decay curves for PCP complexes placed on a glass coverslip and coupled with AgNWs. It is important to note in this regard that when the emission or decay is measured in the latter case, in addition to the PCP complexes coupled strongly with the AgNWs also a substantial contribution of the PCP complexes not affected by the plasmon excitations in AgNWs is included. 

**Figure 5:**
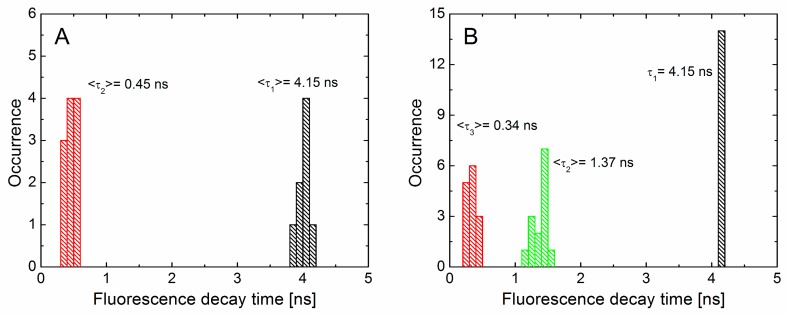
Distribution of decay times constants calculated for PCP complexes away from the nanowires (**A**) and on the nanowires (**B**).

In the case of PCP complexes on the glass coverslip, the decays feature a bi-exponential decay character, which is different from a single exponential curve measured in solution. We attribute this difference to a very thin PVA polymer layer, both the PCP complexes and AgNWs are embedded within. As a result, many of the PCP complexes are exposed to the interfaces with either glass or air, which can modify their fluorescence properties. In Figure 5 we plot histograms of decay constants obtained by fitting the decay curves for PCP off and on AgNWs. In the first case, average values of the decay times were calculated to be 4.15 ns, and 0.45 ns. The distributions of both decay times plotted in Figure 5A are very narrow, indicating rather homogeneous property of the photoactive protein used to monitor the plasmonic interactions with AgNWs. In the first attempt to extract decay time parameters in the case of experiments carried out for PCP complexes deposited in the vicinity of AgNWs, we fixed both times and aimed to reconstruct the decay curves by addition of a third decay constant. This procedure however was not successful. Therefore, we fit these curves by fixing the 4.15 ns component. The decay constants extracted in this way are collected in Figure 5B. Their average values were 0.34 ns and 1.37 ns. This is likely because only the longest, 4.15 ns component is intrinsic to the fluorescence of PCP complexes [33] while the others result from external influences upon the fluorophore. In addition, the observed variability of the shorter decay times can be attributed to heterogeneity of the sample. While the shortest decay time, shown in red in Figure 5, likely originates from exposing high proportion of PCP to glass-polymer and polymer-air interfaces, the intermediate component, shown in green in Figure 5, may result from interactions with the AgNWs. It points out that the increase in fluorescence intensity originates from increase of the spontaneous emission rate of PCP. 

**Figure 6:**
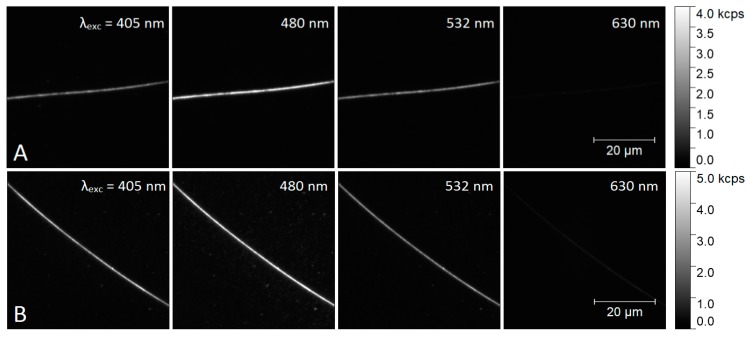
Fluorescence intensity maps of PCP in a PVA layer containing as-synthesized AgNWs (**A**) and aged AgNWs (**B**). Maps were collected for excitation wavelengths of: 405 nm, 480 nm, 532 nm, and 630 nm.

The influence of nanowire morphology on the fluorescence of PCP can be visualized using imaging microscopy. We studied two samples containing PCP and AgNWs, with the exception that in the first sample as-synthesized AgNWs were used, while the second sample contained aged AgNWs. We have collected sets of fluorescence intensity maps for four excitation wavelengths: 630 nm, 532 nm, 480 nm, and 405 nm in this exact order, with the detection wavelength set to 670 ± 5 nm. Such an order, defined by increasing photon energy, reduces photobleaching effect. Overall, 3 sets for maps containing 5 nanowires were collected for the sample with as-synthesized AgNWs, and 5 sets with 9 nanowires were collected for the sample with aged AgNWs. Selected sequences are displayed respectively in Figure 6A,B. 

On both sequences shown in Figure 6 we find long, bright lines, whose positions correlate with the positions of the nanowires seen in transmission images taken of the same sample areas. These bright lines can be then attributed to the emission of PCP complexes located near the AgNWs and strongly coupled with AgNW plasmon resonances. The nanowires are typically much longer than the size of the area imaged in a single experiment, thus the sequences contain just segments of nanowires. The increase of fluorescence emission of PCP complexes is found for all excitation wavelengths. Importantly, the PCP emission is also detected off the nanowires, but its intensity is much lower. 

**Figure 7:**
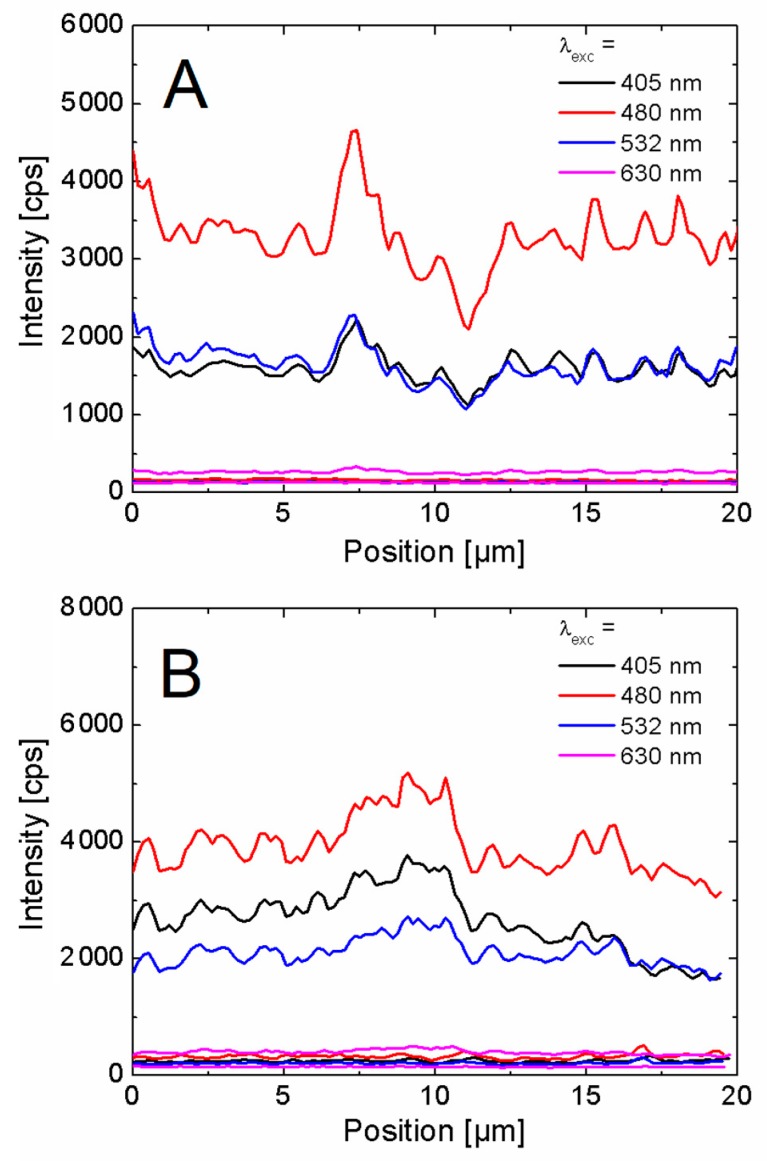
Fluorescence intensity profiles extracted for as-synthesized (**A**) and aged (**B**) AgNWs obtained for the excitation wavelength for PCP located on the nanowire and off the nanowire.

Qualitative comparison between the fluorescence maps detected for as-synthesized and aged AgNWs suggests that the distribution of PCP fluorescence intensity is much more uniform in the case of the latter. We analyzed this effect by extracting fluorescence intensity along AgNWs for all four excitation wavelengths. As a reference stripes of identical length from the areas free of AgNWs were analyzed. Figure 7A,B show plots of fluorescence intensity along as-synthesized and aged AgNW, respectively, for all four excitation wavelengths. 

Fluorescence intensities of PCP on AgNWs are considerably higher than for the reference. Indeed, the emission of PCP off AgNWs is around 100 counts, while for PCP coupled with plasmon excitations in AgNWs counts of a few thousands are measured. In previous experiments the fluorescence enhancement of up to 4-fold was reported [3,4], which is much less than measured for our structures. This is likely due to a very thin PVA layer, and the resulting much higher fraction of PCP complexes placed near AgNW coupled to the plasmon resonances. In this respect, the results of wide-field fluorescence imaging are in qualitative agreement with the findings of confocal fluorescence experiment. 

Furthermore, in the case of the as-synthesized AgNWs (Figure 7A) average fluorescence intensity of PCP along the nanowire varied between 300 to 3500 cps for the excitation wavelength of 480 nm. In contrast, for the aged nanowires the variation was much lower with the average values spanning from 1000 to 4000 cps. 

The cross-sections displayed in Figure 7A feature substantial variation in the emission intensity along the as-synthesized AgNW, with the pattern being rather independent on the excitation wavelength. On the other hand, for the aged AgNW (Figure 7B) the variation is much less, which can be attributed to the thin smooth PVP capping layer, as visible in Figure 2B. The variations of fluorescence intensity can be attributed either to local changes in the concentration of PCP complexes on AgNWs, or to variation of plasmonic coupling due to different distance between PCP complexes and AgNWs. While the first possibility concerns primarily the aged AgNWs, for the as-synthesized ones both effects are combined and superimposed. It can also be argued that part of the effects observed experimentally, could be related to interactions between polymers (PVP and PVA) and the protein. While the chemical structures of these compounds prevent any specific interactions between the polymers and the protein, we cannot exclude some unspecific interaction. This could be possible due to the presence of free pairs of electrons on oxygen and nitrogen atoms in polymers and hydrogen from PCP. Nevertheless, all of the experimental results can be coherently explained without invoking any of such unspecific effects or speculating about their nature. 

**Figure 8:**
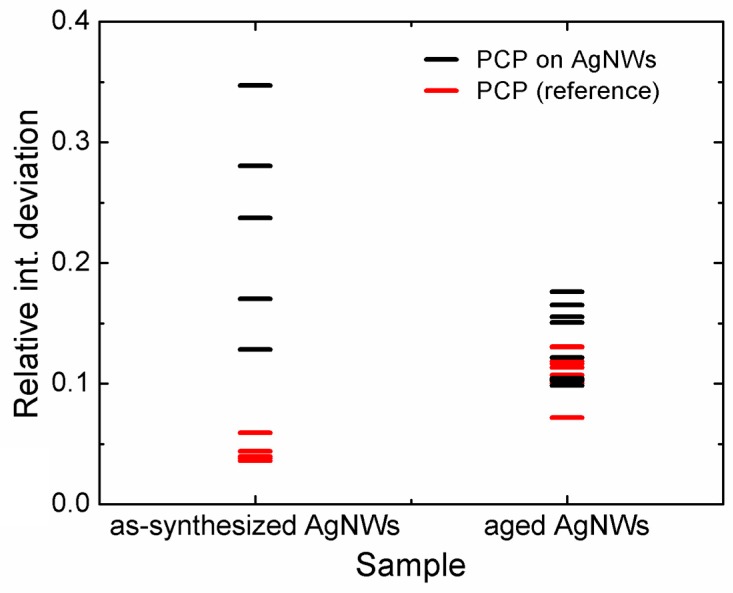
Distribution plot of relative PCP fluorescence intensity deviations (standard deviation divided by mean intensity value along a nanowire) acquired for as-synthesized AgNWs (left) and for aged AgNWs (right). Black dash lines denote fluorescence of PCP in the presence of AgNWs, red lines correspond to PCP-only profiles.

We quantify the variation of fluorescence intensity along AgNWs using a ratio of standard deviation and mean intensity values. Standard deviations were calculated for all points along a fluorescence intensity profile similar to that shown in Figure 7, and then divided by the value of an average intensity of the same profile. The values of these ratios (relative intensity deviations) are plotted in Figure 8 for the samples containing as-synthesized and aged AgNWs. The results obtained for the as-synthesized AgNWs are much more spread indicating inhomogeneous thickness of the PVP capping layer, translating into variations of plasmonic interactions between PCP complexes and AgNWs. 

## 4. Conclusions

Long AgNWs were synthesized using hydrothermal method, with H_2_O_2_ as the reducing agent. The maximum lengths of the nanowires reach 800 µm, keeping at the same time the diameters in the range of 100 nm, thus much less than the wavelength of the visible light. The results of fluorescence imaging and spectroscopy obtained for a hybrid nanostructure composed of photosynthetic complexes and AgNWs indicate a crucial role of the morphology of the metallic nanostructure both on the scale and uniformity of emission enhancement. The results point towards the importance of the morphology of plasmonically active AgNWs when considering their application in enhancing the optical properties or achieving energy propagation over submillimeter distances.
